# The Role of Natural Killer Cells in Multiple Sclerosis and Their Therapeutic Implications

**DOI:** 10.3389/fimmu.2013.00063

**Published:** 2013-03-13

**Authors:** Coralie Chanvillard, Raymond F. Jacolik, Carmen Infante-Duarte, Ramesh C. Nayak

**Affiliations:** ^1^Institute of Medical Immunology, Experimental and Clinical Research Center, Charité – Universitätsmedizin Berlin, A Joint Cooperation Between the Charité, Universitätsmedizin Berlin and the Max-Delbrück Center for Molecular MedicineBerlin, Germany; ^2^MSDx Inc.Tucson, AZ, USA

**Keywords:** natural killer cells, multiple sclerosis, autoimmunity, immunopathogenesis, disease-modifying therapy

## Abstract

Multiple sclerosis (MS) is assumed to be an autoimmune disease initiated by autoreactive T cells that recognize central nervous system antigens. Although adaptive immunity is clearly involved in MS pathogenesis, innate immunity increasingly appears to be implicated in the disease. We and others have presented evidence that natural killer (NK) cells may be involved in immunoregulation in MS, leading to the question of whether a particular NK cell subtype will account for this effect. Changes of NK cell functionality in MS were associated with MS activity, and depletion of NK cells exacerbated the course of disease in a murine model of MS, experimental autoimmune encephalomyelitis. Several studies described a deficiency and transient “valleys” in NK cell killing activity in human MS, which may coincide with symptomatic relapse. However, the molecular basis of the defect in killing activity has not been determined. We discuss results on the expression of perforin in CD16^+^ NK cells and the existence of an inverse relationship between myelin loaded phagocytes and the proportion of CD16^+^ NK cells expressing perforin in the circulation. This inverse relationship is consistent with a role for NK cell killing activity in dampening autoimmunity. On the other hand, it has been broadly reported that first line MS therapies, such as interferon-beta, glatiramer acetate as well as escalation therapies such as fingolimod, daclizumab, or mitoxantrone seem to affect NK cell functionality and phenotype *in vivo*. Therefore, in this review we consider evidence for the immunoregulatory role of NK cells in MS and its animal models. Furthermore, we discuss the effect of MS treatments on NK cell activity.

## Introduction

Multiple sclerosis (MS) is the most common autoimmune disease of the central nervous system (CNS) leading to severe disability in young adults. It is considered to be initiated by autoreactive T cells that recognize CNS antigens and, in concert with numerous immune cells, orchestrate an inflammatory reaction which eventually results in demyelination and neuroaxonal damage (Aktas et al., 2007). Recently, the role of innate immunity in the pathogenesis of MS has attracted more attention and studies on the role of natural killer (NK) cells are appearing in the literature (Gandhi et al., 2010).

**Figure 1:**
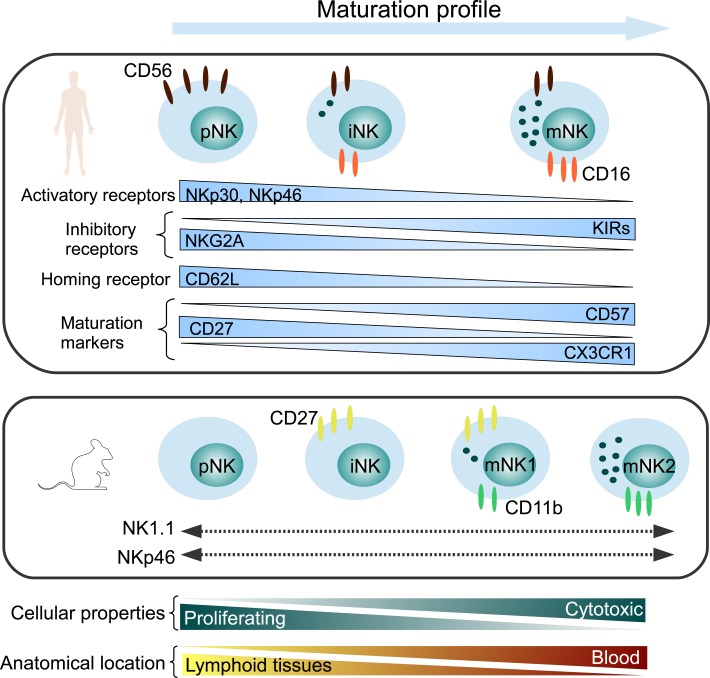
**Maturation profile of human and murine NK cells, representing major NK cell markers**. pNK, precursor NK cells; iNK, immature NK cells; mNK, mature NK cells.

It is widely accepted that NK cells have two key functions, namely cytotoxicity and cytokine secretion and are considered to be mediated by two major NK cell subsets that can be phenotypically distinguished by the expression of CD56 and CD16 antigens in humans (Ferlazzo and Munz, 2004; Strowig et al., 2008). More recently, further markers were shown to distinguish between different maturation subsets. We showed that CX3CR1^−^ NK cells were immature NK cells and that the chemokine receptor CX3CR1 may serve in combination with CD56, CD57, CD62L, and CD27 to define different steps of NK cell maturation (Hamann et al., 2011). The NK cell maturation and receptor expression profile is depicted in Figure 1.

CD56^dim^CD16^+^ “mature” NK cells represent about 90% of total peripheral blood NK cells and are efficient killer cells which are able to secrete variable levels of interferon (IFN)-γ depending on the stimuli. CD56^bright^ CD16^−^ “immature” NK cells constitute less than 10% of total blood NK cells and are able to produce large amounts of cytokines upon stimulation, but develop cytolytic activity only after prolonged stimulation (Strowig et al., 2008). These “immature” NK cells are however enriched in secondary lymphoid tissues (Ferlazzo et al., 2004) and sites of inflammation (Dalbeth et al., 2004). Interestingly in the neuron-immunologic context of MS, we recently discovered that contrary to blood NK cells, NK cells trafficking to the CSF display a phenotype characteristic for a very early stage of maturation (Hamann et al., 2012).

## NK Cells in the Pathogenesis of Experimental Allergic Encephalomyelitis

Experimental Allergic Encephalomyelitis (EAE) is an induced animal model that mimics some features of MS (Constantinescu et al., 2011), therefore providing potentially useful insights into the pathogenesis of this disease. For instance, the potential for treating MS with blocking antibodies to the adhesion molecule VLA4 was first demonstrated in the EAE model and has ultimately resulted in the extremely effective disease-modifying MS drug natalizumab (discussed in more detail below).

It is well established that EAE is induced by CD4^+^ T cells specific for myelin antigens. However, demyelination is a result of complex interactions among encephalitogenic, regulatory, and accessory cell populations and factors produced by these cells. The outcome of the disease depends on which components become dominant. Animal studies have clearly shown that EAE is regulated in a complex way, and that a given component can have very different effects depending on the local microenvironment in which it acts (Morandi et al., 2008). This may explain why NK cells have been reported to both increase and decrease the severity of EAE.

Shi et al. (2000) demonstrated that MOG failed to induce EAE in IL-18 knock-out mice. Features of EAE could be restored upon IL-18 administration but disease required the presence of IFN-γ-producing NK cells. In contrast, Zhang et al. (1997) have reported that *in vivo* NK cell depletion resulted in exacerbation of clinical symptoms in wild-type C57BL/6 mice. In addition, they also found that depletion of NK cells resulted in an increased severity of symptoms when disease was induced by passive transfer of a MOG-specific T cell line. Furthermore, Xu et al. (2005) reported that EAE is exacerbated by NK cell depletion and that NK cells in PLP induced EAE exert a direct cytotoxic effect on autoantigen-specific, encephalitogenic T cells. Moreover, NK cells expansion (after IL-2 monoclonal antibody complexes injection) attenuated EAE and reduced production of CD4+ Th17 in the CNS (Hao et al., 2011). The observations of Xu et al. may be particularly relevant to the pathogenesis of MS.

## NK Cells in the Pathogenesis of Multiple Sclerosis

The vast majority of studies on the immunopathogenesis of MS have focused on the role of T cells. However, research reports spanning more than three decades have established that there is a significant role of NK cells in relapsing–remitting MS (RRMS) patients (Benczur et al., 1980; Merrill et al., 1982; Neighbour et al., 1982; Oger et al., 1988; Kastrukoff et al., 1998, 2003; Infante-Duarte et al., 2005; Hamann et al., 2012). Activated NK cells are capable of cytolysis of autologous oligodendrocytes and are found in acute inflammatory lesions. In MS however, most studies report a deficiency of NK cytolytic activity in peripheral blood (Benczur et al., 1980; Merrill et al., 1982; Neighbour et al., 1982; Oger et al., 1988; Kastrukoff et al., 1998, 2003). These studies employ the *in vitro* chromium^51^ release assay using K562 tumor cells as the target. In 1980, Benczur et al. (1980) reported that NK cytolytic activity against K562 targets was significantly low in MS patients and most particularly so in male patients with a definitive diagnosis of MS. This finding of diminished NK cell-mediated cytotoxicity was quickly reproduced (Merrill et al., 1982; Neighbour et al., 1982).

Oger et al. (1988) performed a small longitudinal study and found that patients with large asymptomatic MRI lesions had reduced NK cytolytic activity which subsequently increased as the MRI lesions diminished. Moreover, we reported an elevated frequency of immature circulating CX3CR1^−^ NK cells in stable but not in active MS patients (Infante-Duarte et al., 2005) supporting the role of particular NK cell subsets in mediating relapse and remission. Furthermore, Kastrukoff et al. (1998, 2003) have performed longitudinal studies of NK cytolytic activity in RRMS subjects and have demonstrated “valleys” in killing activity that last 4–5 weeks. These transient deficits in cytolytic activity may explain why some studies failed to detect diminished NK cytolytic activity. More important however is their finding of a significant correlation between valleys in NK cell killing activity and new or enlarging active lesions on MRI as well as with clinical exacerbations. These authors concluded that valleys in NK cell killing activity represent periods of susceptibility for the development of active lesions on MRI and clinical attacks and that these valleys are the result of cells with an NK phenotype being unable to deliver a “lethal” hit to targets (Kastrukoff et al., 2003).

These studies fit well with reports that NK cell depletion exacerbates disease activity in EAE in rodents.

## Effects of Current and Emerging MS Therapies on NK Cells

Since the first documented case of MS in the nineteenth century, treatments have evolved in parallel with the emerging understanding of the disease. In the 1860s, Dr. Charcot’s trial to treat MS patients with gold and silver proved unsuccessful (Clanet, 2008). Over 150 years later, the spectrum of MS therapies has evolved to target the major immunopathological players of the disease, T and B cells (Bar-Or, 2008). However, our comprehension of MS is still far from complete and further immunological aspects of the disease, such as involvement of innate immunity and in particular NK cells, have begun to arouse interest.

Although there is still no cure for MS, a range of disease-modifying therapies are available for treating patients. These therapies diminish the risk of relapse but exert limited effects in delaying the progression of disability (Lim and Constantinescu, 2010). Despite the broadening spectrum of available therapies for MS in the past few years, there nevertheless remains a lack of selectivity in the available treatments, leading to serious side effects. Currently, the principal approved disease-modifying therapies include IFN-β, glatiramer acetate (GA), natalizumab, fingolimod, mitoxantrone (Lim and Constantinescu, 2010), and the very recently approved teriflunomide (Nordqvist, 2012). MS immunomodulatory therapies principally benefit RRMS patients, but have been revealed to be ineffective for patients with progressive forms of the disease. Moreover, several new therapies with a variety of actions are currently being tested in phase III clinical trials, including the oral treatments dimethyl fumarate (NCT00420212) and laquinimod (NCT01047319); and the monoclonal antibodies daclizumab (NCT01064401), rituximab (NCT00087529), and alemtuzumab (NCT00548405).

Natural Killer cells are being recognized as a hitherto unexplored player in MS pathogenesis. Thus, the investigation of how different treatments influence NK cell immunology is becoming important in understanding drug-related mechanisms of action and for dissecting out the involvement of NK cells and their subsets in autoimmune neuroinflammation. The principal MS therapies and their modes of action are summarized in Table 1.

**Table 1:** **Overview of the effect of current and emerging MS therapies on NK cells**.

Treatment	Mode of action	Effects on NK cells	Induced NK cell phenotype
**APPROVED THERAPIES**			
IFN-β	Broad	Decrease of total circulating NK cells in MS patients	Immature
		Expansion of immunoregulatory CD56^bright^/decrease of the cytotoxic CD56^dim^ NK cells in the periphery (Perini et al., 2000; Saraste et al., 2007; Vandenbark et al., 2009; Martinez-Rodriguez et al., 2010)	
Glatiramer acetate	APC-P	Influence dendritic cell susceptibility to promote NK cell cytotoxicity (Al-Falahi et al., 2009; Sand et al., 2009)	unk.
Mitoxantrone	IS	Indirect NK cell enrichment, due to the depletion of other lymphocyte population such as B cells	Mature
		NK cell maturation only in responders to treatment (Chanvillard et al., 2012)	
Natalizumab	TI	MS: increase of total NK cells and CD56^bright^ NK cells in blood (Putzki et al., 2010; Skarica et al., 2011)	Immature
		EAE: treatment reduced NK cell numbers in spleen, lymph nodes, and CNS, comparing with non-treated mice (Gan et al., 2012)	
		Treatment broad impact on NK cell trafficking (Gan et al., 2012)	
Fingolimod	TI	Diverging results on total NK cells in circulation	Mature
		Decreased CD56^bright^ CD62L^+^ NK cell proportions in blood	
		Lymph node egress of immature NK cells blocked (Johnson et al., 2011)	
		Increased NK cell numbers in CSF of treated patients (Kowarik et al., 2011)	
Teriflunomide	AM	Peripheral NK cell expansion from abnormally low initial values in RA patients (Manda et al., 2009)	unk.
**ON-GOING TRAILS**			
Daclizumab	LD	Higher bioavailability of IL-2 cytokine leading to a large expansion of the NK cell population (Martin et al., 2010; Hao et al., 2011)	Immature
		Expansion of the CD56^bright^ NK cell in the blood, correlating with a positive response to the treatment (Bielekova et al., 2009)	
		Expansion of CD56^bright^ NK cells in the CSF of treated patients (Bielekova et al., 2011)	
Alemtuzumab	LD	Pivotal role of ADCC by NK cells in tumor treatments using monoclonal antibodies	Immature
		Depletion of B and T lymphocytes from the circulation of alemtuzumab treated mice. Cells with little expression of CD52 such as mature NK cells were comparably less affected (Hu et al., 2009)	
		Alemtuzumab activity ablated by NK cell depletion, indicating a prominent role for NK cell-mediated ADCC in lymphocyte depletion	
Rituximab	BD	ADCC by NK cells previously described to be an essential therapeutic mechanism of rituximab in lupus treatment (Anolik et al., 2003)	
		Rituximab clinical outcome in cancer associated with a polymorphism inducing a variation of the FcgRIII receptor affinity (Veeramani et al., 2011)	

*unk., unknown; APC-P, antigen-presenting cell promotion; IS, immunosuppressive; TI, T cell inhibition; AM, antimetabolites; LD, leukocyte depletion; BD, B cell depletion*.

## Effects of Approved MS Therapies on NK Cells

### First line therapies: IFN-β and GA

Interferon-β has become a worldwide standard in the treatment of MS. It has immunomodulatory properties, and has been shown to inhibit T cell proliferation, and to impair the trafficking of inflammatory cells into the CNS (Yong, 2002; Lim and Constantinescu, 2010). IFN-β appears to induce a slight decrease of total circulating NK cells in MS patients. Moreover, several studies have shown a marked expansion in the proportion of the immunoregulatory NK cell subset CD56^bright^ coupled with a decrease of the cytotoxic CD56^dim^ NK cells in the periphery following IFN-β therapy (Perini et al., 2000; Saraste et al., 2007; Vandenbark et al., 2009; Martinez-Rodriguez et al., 2010). IFN-β is a potent antiviral mediator. Thus, the expansion of CD56^bright^ NK cells by IFN-β might be related to the enhancement of NK cell functions following the antiviral status induced by this therapy (Kastrukoff et al., 1999; Hartrich et al., 2003). It has been reported that expansion of CD56^bright^ NK cell and of the inhibitory NK cell receptor NKG2A in blood in MS patient, as well as reduction of both NK cells markers LILRB1 and KIR could be linked to IFN-β treatment, and more specifically to positive clinical outcome (Martinez-Rodriguez et al., 2010, 2011). These results suggest that enhancement of NK cell activity may represent an additional mechanism of action of IFN-β in MS (Martinez-Rodriguez et al., 2011). Altogether, these findings suggest that NK cell maturation phenotype is related to the therapeutic benefit of IFN-β, although NK cell could not yet be used as biomarker for response to this therapy.

Glatiramer acetate is a random synthetic polymer composed of four amino acids that are found in myelin basic protein. GA has been reported to promote the development of antigen-presenting cells showing anti-inflammatory properties, such as monocytes (Weber et al., 2007) and dendritic cells (Moretta, 2002). Since bilateral actions between antigen-presenting cells and NK cells have been demonstrated (Ferlazzo et al., 2002; Moretta, 2002; Degli-Esposti and Smyth, 2005), it has been hypothesized that GA modulates NK cell functional via antigen-presenting cells. In the animal model of MS, GA-treated mice displayed a higher killing efficiency of their NK cells against dendritic cells (Al-Falahi et al., 2009). However, GA did not have a direct effect on NK cells activation (Al-Falahi et al., 2009). Similarly, GA enhanced NK cell cytolysis against dendritic cells in MS patients, without affecting levels of NK cell activating markers such as NKG2D, NKp30, or NKp44 (Sand et al., 2009). Thus, GA seems to influence dendritic cell susceptibility to NK cell cytotoxicity.

### Second line therapies: Mitoxantrone, natalizumab, fingolimod, and teriflunomide

Thus far, only a very limited number of treatments are available for patients with progressive MS. The potent immunosuppressive agent mitoxantrone is one of these. However, its use is limited by the appearance of severe side effects. Apart from its general cytotoxic properties, the mode of action of mitoxantrone on the immune system is poorly understood. We have recently shown that mitoxantrone treatment promoted an indirect NK cell enrichment, due to the depletion of other lymphocyte population such as B cells. Importantly, we have discovered that prolonged treatment with mitoxantrone induced NK cell maturation, occurring only in those patients that showed a clinical response to treatment (Chanvillard et al., 2012).

Natalizumab was designed to prevent leukocyte accumulation in the CNS of EAE (Kent et al., 1995; Yednock et al., 1995), and is the first humanized monoclonal antibody approved for treatment of the relapsing–remitting form of MS (Miller et al., 2003). Natalizumab is directed against the very-late antigen-4 integrin, which has a crucial role in the transmigration of immune cells across the blood–brain barrier (Ransohoff, 2007). Following natalizumab treatment, total NK cells (Putzki et al., 2010; Planas et al., 2012) as well as CD56^bright^ NK cells were reported to increase in peripheral blood of patients (Skarica et al., 2011). In EAE, Gan and colleagues reported that natalizumab treated mice had lower numbers of NK cells in the peripheral lymphatic system (spleen and lymph nodes) and in the CNS, compared to non-treated mice. These findings suggest that natalizumab may exert a broad impact on the trafficking of NK cells (Gan et al., 2012). Thus, the increase in circulating CD56^bright^ NK cells in MS patients could be due to NK cell migration from the lymphoid organs, where the CD56^bright^ subset is mainly present, to the circulation. Likewise, their entry into the CNS seems to be blocked. Whether such altered distribution of NK cells has an impact on the therapeutic effects of natalizumab remains unknown.

Fingolimod is an oral compound approved for the treatment of MS. It is a sphingosine 1-phosphate receptor agonist with demonstrated efficacy in reducing inflammatory events in the CNS of MS patients (Kappos et al., 2006). Upon phosphorylation *in vivo*, fingolimod rapidly inhibits lymphocyte egress from lymph nodes, therefore preventing access of activated autoreactive T cells to the CNS. Interestingly, despite divergent results on total NK cells in circulation (Vaessen et al., 2006; Kowarik et al., 2011), fingolimod treatment decreased the proportions of circulating CD56^bright^CD62L^+^ NK cells, without modulating its capacity to produce IFN-γ, tumor necrosis factor (TNF)-α, and IL-10. Within the lymph nodes, about 90% of the NK cells display an immature CD56^bright^CD62L^+^ phenotype. Thus similarly to the action on T cells, fingolimod seems to block the egress of immature NK cells from the lymph nodes (Johnson et al., 2011). Moreover, fingolimod was found to increase NK cell numbers in CSF of treated patients (Kowarik et al., 2011).

Teriflunomide, the active compound of leflunomide, is an oral drug that was very recently approved by the FDA for MS treatment in the USA. Leflunomide is licensed for the treatment of rheumatoid arthritis and is also effective in experimental autoimmune neuritis and EAE (Korn et al., 2001, 2004). Although few studies have been reported in MS so far, peripheral NK cells have been shown to expand from abnormally low initial values in RA patients (Manda et al., 2009).

## Effects of Emerging MS Therapies on NK Cells

Several immunomodulatory therapies are currently being tested for their safety and efficacy in controlling brain inflammation and preventing disease progression in MS.

### Daclizumab

Daclizumab is a humanized monoclonal antibody currently in phase III clinical trials for MS. Daclizumab significantly inhibits the appearance of lesions and improve clinical scores (Bielekova et al., 2004, 2009). This treatment was originally given to block the high affinity IL-2 receptor subunit (CD25), a crucial component of T cell proliferation and activation. The action of daclizumab has also been related to a higher bioavailability of IL-2 cytokine, unexpectedly leading to a large expansion of the NK cell population (Martin et al., 2010; Hao et al., 2011). Daclizumab treatment was reported to induce an expansion of the CD56^bright^ NK cell subset in the blood of MS patients (Bielekova et al., 2006). Moreover, this increase correlated with a positive response to the treatment (Bielekova et al., 2009). The expansion of circulating CD56^bright^ NK cells was associated with decreased brain lesions (Wynn et al., 2010) and inflammation and reduced survival of activated T cells (Bielekova et al., 2004, 2006, 2011). Interestingly, it was recently shown that daclizumab treatment led to a significant expansion of CD56^bright^ NK cells also in the CSF of treated patients, suggesting that NK cells can suppress immune responses directly in the CNS (Bielekova et al., 2011). NK cells, along with lymphoid tissue-inducer cells (LTi), were described to belong to the innate lymphoid cells (ILCs), a novel family of developmentally related hematopoietic effectors that serve protective roles in innate immune responses (Spits and Di Santo, 2011; Spits et al., 2013). Recently, daclizumab therapy was reported to shift ILCs precursors away from LTi phenotype and toward an NK cell lineage. Mechanistic studies indicated that daclizumab inhibited differentiation of LTi indirectly, directing their differentiation toward CD56^bright^ NK cells through enhanced intermediate-affinity IL-2 signaling (Perry et al., 2012).

The specific expansion of CD56^bright^ NK cells in contrast to the CD56^dim^ subset could be explained by their relative expression of the intermediate-affinity IL-2 receptor: CD122. Indeed, this IL-2 receptor is expressed at least 10-fold more by the CD56^bright^ subset compared to CD56^dim^ cells (Bielekova et al., 2006). Therefore, this high expression of intermediate-affinity was hypothesized to allow CD56^bright^ NK cells to sustain their IL-2 signaling in the presence of daclizumab, leading to their proliferation (Martin et al., 2010; Bielekova, 2013).

Thus, while therapies such as GA or IFN-β do not primarily target NK cells but have effects on multiple immune cell types, it appears that expansion of the regulatory CD56^bright^ population of NK cells via intermediate IL-2 receptor signaling is an important biological effect of daclizumab treatment (Kala et al., 2011; Kieseier and Stuve, 2011; Martin, 2012).

### Further monoclonal antibody therapies, alemtuzumab, and rituximab

Natural Killer cells were shown to mediate a lytic attack (antibody-dependent cellular cytotoxicity, ADCC) through binding to FcγRIII receptor (CD16) (Perussia et al., 1984). Several *in vivo* and clinical studies assessed the pivotal role of ADCC by NK cells in tumor treatments using monoclonal antibodies (Clynes et al., 2000). Alemtuzumab is a humanized monoclonal antibody against CD52, a surface antigen of normal and malignant lymphocytes. It is approved for the treatment of B cell chronic lymphocytic leukemia and is undergoing phase III clinical trials for MS. Treatment of mice with this substance results in dose-dependent depletion of B and T lymphocytes from the circulation. Cells with little expression of CD52 such as mature NK cells were comparably less affected. Curiously, removal of NK cells essentially ablated alemtuzumab activity, suggesting a prominent role for NK cell-mediated ADCC in lymphocyte depletion by this therapy (Hu et al., 2009). Also, rituximab is a chimeric monoclonal antibody, which targets the CD20 antigen on pre B cells and B cells. Although NK cells have not been investigated in MS during this treatment, ADCC by NK cells was previously described to be an essential therapeutic mechanism of rituximab in lupus treatment (Anolik et al., 2003). Moreover, rituximab clinical outcome in cancer was associated with a polymorphism inducing a variation in receptor affinity of the FcγRIII (Dall’Ozzo et al., 2004; Veeramani et al., 2011).

## Regulation of Autoimmunity by NK Cells in Multiple Sclerosis

Regulation of autoimmunity by NK cells can occur through secretion of cytokines and the effect of those cytokines in the local milieu or through cytolytic deletion of effector cells such as T cells and/or antigen-presenting cells.

Disease-modifying therapies for MS that have an effect on NK cells tend to increase the CD56^bright^ CD16^−^ “immature” NK cell population in peripheral blood. In contrast, very little is known about the role of NK cell cytolytic activity in dampening autoimmunity in MS. While published studies have demonstrated that there are transient cyclical deficits in NK cell cytolytic competence as assessed by functional assay, the targets *in vivo* are only just beginning to be elucidated. As mentioned earlier, dendritic cells may be targets of NK cell cytotoxicity designed to dampen autoimmunity by editing antigen presentation, at least when treated with GA.

Nielsen et al. (2012) have studied the ability of CD56^bright^ and CD56^dim^ NK cells to kill autologous activated CD4^+^ T cells through the granule exocytosis pathway. They found that both subsets efficiently killed activated, but not resting, CD4+ T cells. Degranulation of NK cells toward activated CD4+ T cells was enhanced by IL-2, IL-15, IL-12 + IL-18, and IFN-α, however, IL-7 and IL-21 stimulated degranulation by CD56^bright^ NK cells but not by CD56^dim^ NK cells.

Recently, Jiang et al. (2011) have demonstrated that CD56^bright^ NK cells can lyze activated T cells through a granzyme K mediated mechanism. These NK cells degranulate to cause caspase independent apoptosis and mitochondrial dysfunction in activated T cells by preferential transfer of granzyme K. Gene silencing of granzyme K with siRNA abrogated the ability of a CD56^bright^ human NK cell line to kill syngeneic activated T cells. These authors also showed that treatment of MS patients with daclizumab significantly enhanced this mechanism of cytotoxicity by CD56^bright^ NK cells.

The evidence for NK cell regulation of autoimmune effector cells by cytolytic deletion in MS is sparse but intriguing and will undoubtedly provoke more studies. The deficit of NK killing activity in MS has not been explained at the molecular level. As cytolysis of target cells by NK cells is mediated by one of two distinct molecular mechanisms, the death receptor pathway and the granule exocytosis pathway, it could be expected that molecular components of either pathway may be involved.

The death receptor pathway is mediated by membrane-bound or soluble factors belonging to the TNF superfamily that interact with one of the membrane-bound TNF-receptor (TNFR) superfamily agents (MacEwan, 2002). Trimerization of TNFRs [e.g., TNFR1, Fas, and TNF-related apoptosis-inducing ligand (TRAIL) receptors] activates death-domains in their intracellular tails, which leads to activation of caspases and cell death. The granule exocytosis pathway is dependent on the pore-forming protein perforin, which allows passage of serine proteases (granzymes) into the cytoplasm of target cells (Catalfamo and Henkart, 2003). Granzymes induce cell death either through caspase dependent apoptosis or mitochondrial injury.

At the 2012 meeting of the Society for Natural Immunity, we presented experimental results from a cross sectional study indicating that untreated MS patients had a relative deficiency of CD16^+^Perforin^+^ NK cells relative to apparently healthy controls (Nayak and Jacolik, 2012). A threshold level of CD16^+^/Perforin^+^ lymphocytes was found at 75%. Ninety one percent of apparently healthy controls had greater than 75% CD16^+^/perforin^+^ lymphocytes in comparison with 50% of untreated MS subjects. CD16^+^/perforin^+^ lymphocytes in glatiramer acetate treated MS subjects were not statistically different from apparently healthy controls, a finding that is consistent with the study reported by Sand et al. (2009) demonstrating increased killing by GA-treated NK cells. No difference in CD16^+^/Perforin^+^ lymphocytes was seen in stroke patients. Low levels of CD16^+^/Perforin^+^ lymphocytes has not yet been correlated with the killing deficit in MS but could potentially begin to provide an explanation for this deficit in MS.

## Concluding Remarks

The role of NK cells in dampening autoimmunity and the mechanisms by which this is achieved is of particular interest from a therapeutic point of view. The differential effects of cytokines such as IL-7 and IL-21 in stimulating granule dependent killing in NK cell subpopulations hints at the complexity of regulation of NK cell-mediated cytotoxicity and the potential importance of tissue specific microenvironments in determining functional activity. Of particular importance may be the generation of transient changes in cytokine profiles that may lead to temporary deficits in NK cell killing activity that has been reported to correlate with clinical exacerbations and radiological disease activity in MS. The current disease-modifying therapies have been developed to have a salutary effect on clinical endpoints and so the mechanisms of action remain an object of study after they are in clinical use. Many of the current disease-modifying therapies for MS do have effects on NK cells. However, as more becomes known about how NK cells are regulated in normal and diseased tissues, therapeutic development may specifically target processes that regulate NK cell functions. For example, compounds that increase the proportion of CD16^+^Perforin^+^ NK cells may prevent disease relapses and targeted expression of IL-7 and CD21 may provide competency signals that convert CD56^bright^ NK cells from a predominantly cytokine producing cell type to an efficient cytotoxic cell type. This may also be particularly beneficial in tumors where infiltrating NK cells are predominantly CD56^bright^ cytokine producing cells (Carrega et al., 2008). In recent years, NK cells have emerged as potential biological markers for the response to different MS therapies, including IFN-β, mitoxantrone, and daclizumab. Notably, the immunoregulatory and immature NK cell subset CD56^bright^ seems to play an important role in the resolution of MS and to mediate the beneficial effects of several therapies, potentially via modulation of T cell responses. A paradox rises between treatments promoting a more immature NK cell phenotype in the periphery, such as IFN-β, natalizumab, and daclizumab, also linked to a positive clinical outcome; and those inducing a more mature phenotype, such as mitoxantrone and fingolimod.

The increased number of CD56^bright^ NK cells after treatment could result from a diminished maturation rate of CD56^bright^ toward CD56^dim^ NK cells, as those have been described by Romagnani et al. (2007) as being able to maturate into CD56^dim^ cells upon activation. Further possible mechanisms of CD56^bright^ expansion include differential homing of NK cell populations following treatment, leading to an increased output of immature CD56^bright^ NK cells from lymphoid organs into the peripheral circulation. Although trafficking of NK cells has been extensively studied in the mouse (Gregoire et al., 2007), human data are scarce (Carrega and Ferlazzo, 2012) and so far, only speculative.

Taken together, these studies highlight the importance of the regulatory role of NK cells in the immunopathology of MS. Moreover, the precise beneficial NK cell phenotype that should be targeted in MS remains to be determined.

## Conflict of Interest Statement

Ramesh C. Nayak and Raymond F. Jacolik are employees of MSDx Inc. and own shares in MSDx Inc.

## References

[B1] Aktas O. Ullrich O. Infante-Duarte C. Nitsch R. Zipp F. (2007). Neuronal damage in brain inflammation. Arch. Neurol. 64, 185–189 10.1001/archneur.64.2.185 17296833

[B2] Al-Falahi Y. Sand K. L. Knudsen E. Damaj B. B. Rolin J. Maghazachi A. A. (2009). Splenic natural killer cell activity in two models of experimental neurodegenerative diseases. J. Cell. Mol. Med. 13, 2693–2703 10.1111/j.1582-4934.2008.00640.x 19397784 PMC6529976

[B3] Anolik J. H. Campbell D. Felgar R. E. Young F. Sanz I. Rosenblatt J. (2003). The relationship of FcgammaRIIIa genotype to degree of B cell depletion by rituximab in the treatment of systemic lupus erythematosus. Arthritis Rheum. 48, 455–459 10.1002/art.10764 12571855

[B4] Bar-Or A. (2008). The immunology of multiple sclerosis. Semin. Neurol. 28, 29–45 10.1055/s-2007-1019124 18256985

[B5] Benczur M. Petranyl G. G. Palffy G. Varga M. Talas M. Kotsy B. (1980). Dysfunction of natural killer cells in multiple sclerosis: a possible pathogenetic factor. Clin. Exp. Immunol. 39, 657–662 6155232 PMC1538133

[B6] Bielekova B. (2013). Daclizumab therapy for multiple sclerosis. Neurotherapeutics 10, 55–67 10.1007/s13311-012-0147-4 23055048 PMC3557356

[B7] Bielekova B. Catalfamo M. Reichert-Scrivner S. Packer A. Cerna M. Waldmann T. A. (2006). Regulatory CD56(bright) natural killer cells mediate immunomodulatory effects of IL-2Ralpha-targeted therapy (daclizumab) in multiple sclerosis. Proc. Natl. Acad. Sci. U.S.A. 103, 5941–5946 10.1073/pnas.0601335103 16585503 PMC1458677

[B8] Bielekova B. Howard T. Packer A. N. Richert N. Blevins G. Ohayon J. (2009). Effect of anti-CD25 antibody daclizumab in the inhibition of inflammation and stabilization of disease progression in multiple sclerosis. Arch. Neurol. 66, 483–489 10.1001/archneurol.2009.50 19364933 PMC2742781

[B9] Bielekova B. Richert N. Herman M. L. Ohayon J. Waldmann T. A. McFarland H. (2011). Intrathecal effects of daclizumab treatment of multiple sclerosis. Neurology 77, 1877–1886 10.1212/WNL.0b013e318239f7ef 22076546 PMC3246406

[B10] Bielekova B. Richert N. Howard T. Blevins G. Markovic-Plese S. McCartin J. (2004). Humanized anti-CD25 (daclizumab) inhibits disease activity in multiple sclerosis patients failing to respond to interferon beta. Proc. Natl. Acad. Sci. U.S.A. 101, 8705–8708 10.1073/pnas.0402653101 15161974 PMC423259

[B11] Carrega P. Ferlazzo G. (2012). Natural killer cell distribution and trafficking in human tissues. Front. Immunol. 3:347 10.3389/fimmu.2012.00347 23230434 PMC3515878

[B12] Carrega P. Morandi B. Costa R. Frumento G. Forte G. Altavilla G. (2008). Natural killer cells infiltrating human nonsmall-cell lung cancer are enriched in CD56 bright CD16(-) cells and display an impaired capability to kill tumor cells. Cancer 112, 863–875 10.1002/cncr.23239 18203207

[B13] Catalfamo M. Henkart P. A. (2003). Perforin and the granule exocytosis cytotoxicity pathway. Curr. Opin. Immunol. 15, 522–527 10.1016/S0952-7915(03)00114-6 14499260

[B14] Chanvillard C. Millward J. M. Lozano M. Hamann I. Paul F. Zipp F. (2012). Mitoxantrone induces natural killer cell maturation in patients with secondary progressive multiple sclerosis. PLoS ONE 7:e39625 10.1371/journal.pone.0039625 22768101 PMC3387260

[B15] Clanet M. (2008). Jean-Martin Charcot. 1825 to 1893. Int. MS J. 15, 59–61 18782501

[B16] Clynes R. A. Towers T. L. Presta L. G. Ravetch J. V. (2000). Inhibitory Fc receptors modulate in vivo cytotoxicity against tumor targets. Nat. Med. 6, 443–446 10.1038/74704 10742152

[B17] Constantinescu C. S. Farooqi N. O’Brien K. Gran B. (2011). Experimental autoimmune encephalomyelitis (EAE) as a model for multiple sclerosis (MS). Br. J. Pharmacol. 164, 1079–1106 10.1111/j.1476-5381.2011.01302.x 21371012 PMC3229753

[B18] Dalbeth N. Gundle R. Davies R. J. Lee Y. C. McMichael A. J. Callan M. F. (2004). CD56bright NK cells are enriched at inflammatory sites and can engage with monocytes in a reciprocal program of activation. J. Immunol. 173, 6418–6426 15528382 10.4049/jimmunol.173.10.6418

[B19] Dall’Ozzo S. Tartas S. Paintaud G. Cartron G. Colombat P. Bardos P. (2004). Rituximab-dependent cytotoxicity by natural killer cells: influence of FCGR3A polymorphism on the concentration-effect relationship. Cancer Res. 64, 4664–4669 10.1158/0008-5472.CAN-03-2862 15231679

[B20] Degli-Esposti M. A. Smyth M. J. (2005). Close encounters of different kinds: dendritic cells and NK cells take centre stage. Nat. Rev. Immunol. 5, 112–124 10.1038/nri1549 15688039

[B21] Ferlazzo G. Munz C. (2004). NK cell compartments and their activation by dendritic cells. J. Immunol. 172, 1333–1339 14734707 10.4049/jimmunol.172.3.1333

[B22] Ferlazzo G. Thomas D. Lin S. L. Goodman K. Morandi B. Muller W. A. (2004). The abundant NK cells in human secondary lymphoid tissues require activation to express killer cell Ig-like receptors and become cytolytic. J. Immunol. 172, 1455–1462 14734722 10.4049/jimmunol.172.3.1455

[B23] Ferlazzo G. Tsang M. L. Moretta L. Melioli G. Steinman R. M. Munz C. (2002). Human dendritic cells activate resting natural killer (NK) cells and are recognized via the NKp30 receptor by activated NK cells. J. Exp. Med. 195, 343–351 10.1084/jem.20011149 11828009 PMC2193591

[B24] Gan Y. Liu R. Wu W. Bomprezzi R. Shi F. D. (2012). Antibody to alpha4 integrin suppresses natural killer cells infiltration in central nervous system in experimental autoimmune encephalomyelitis. J. Neuroimmunol. 247, 9–15 10.1016/j.jneuroim.2012.03.011 22503411 PMC3351567

[B25] Gandhi R. Laroni A. Weiner H. L. (2010). Role of the innate immune system in the pathogenesis of multiple sclerosis. J. Neuroimmunol. 221, 7–14 10.1016/j.jneuroim.2009.10.015 19931190 PMC2854189

[B26] Gregoire C. Chasson L. Luci C. Tomasello E. Geissmann F. Vivier E. (2007). The trafficking of natural killer cells. Immunol. Rev. 220, 169–182 10.1111/j.1600-065X.2007.00563.x 17979846 PMC7165697

[B27] Hamann I. Dorr J. Glumm R. Chanvillard C. Janssen A. Millward J. M. (2012). Characterization of natural killer cells in paired CSF and blood samples during neuroinflammation. J. Neuroimmunol. 22948090 10.1016/j.jneuroim.2012.08.009

[B28] Hamann I. Unterwalder N. Cardona A. E. Meisel C. Zipp F. Ransohoff R. M. (2011). Analyses of phenotypic and functional characteristics of CX3CR1-expressing natural killer cells. Immunology 133, 62–73 10.1111/j.1365-2567.2011.03409.x 21320123 PMC3088968

[B29] Hao J. Campagnolo D. Liu R. Piao W. Shi S. Hu B. (2011). Interleukin-2/interleukin-2 antibody therapy induces target organ natural killer cells that inhibit central nervous system inflammation. Ann. Neurol. 69, 721–734 10.1002/ana.22339 21425186 PMC3082615

[B30] Hartrich L. Weinstock-Guttman B. Hall D. Badgett D. Baier M. Patrick K. (2003). Dynamics of immune cell trafficking in interferon-beta treated multiple sclerosis patients. J. Neuroimmunol. 139, 84–92 10.1016/S0165-5728(03)00135-8 12799025

[B31] Hu Y. Turner M. J. Shields J. Gale M. S. Hutto E. Roberts B. L. (2009). Investigation of the mechanism of action of alemtuzumab in a human CD52 transgenic mouse model. Immunology 128, 260–270 10.1111/j.1365-2567.2009.03115.x 19740383 PMC2767316

[B32] Infante-Duarte C. Weber A. Kratzschmar J. Prozorovski T. Pikol S. Hamann I. (2005). Frequency of blood CX3CR1-positive natural killer cells correlates with disease activity in multiple sclerosis patients. FASEB J. 19, 1902–1904 16144955 10.1096/fj.05-3832fje

[B33] Jiang W. Chai N. R. Maric D. Bielekova B. (2011). Unexpected role for granzyme K in CD56bright NK cell-mediated immunoregulation of multiple sclerosis. J. Immunol. 187, 781–790 10.4049/jimmunol.1100361 21666061 PMC3131478

[B34] Johnson T. A. Evans B. L. Durafourt B. A. Blain M. Lapierre Y. Bar-Or A. (2011). Reduction of the peripheral blood CD56(bright) NK lymphocyte subset in FTY720-treated multiple sclerosis patients. J. Immunol. 187, 570–579 10.4049/jimmunol.1003823 21622858

[B35] Kala M. Miravalle A. Vollmer T. (2011). Recent insights into the mechanism of action of glatiramer acetate. J. Neuroimmunol. 235, 9–17 10.1016/j.jneuroim.2011.01.009 21402415

[B36] Kappos L. Antel J. Comi G. Montalban X. O’Connor P. Polman C. H. (2006). Oral fingolimod (FTY720) for relapsing multiple sclerosis. N. Engl. J. Med. 355, 1124–1140 10.1056/NEJMoa052643 16971719

[B37] Kastrukoff L. F. Lau A. Wee R. Zecchini D. White R. Paty D. W. (2003). Clinical relapses of multiple sclerosis are associated with ‘novel’ valleys in natural killer cell functional activity. J. Neuroimmunol. 145, 103–114 10.1016/j.jneuroim.2003.10.001 14644036

[B38] Kastrukoff L. F. Morgan N. G. Zecchini D. White R. Petkau A. J. Satoh J. (1998). A role for natural killer cells in the immunopathogenesis of multiple sclerosis. J. Neuroimmunol. 86, 123–133 10.1016/S0165-5728(98)00014-9 9663557

[B39] Kastrukoff L. F. Morgan N. G. Zecchini D. White R. Petkau A. J. Satoh J. (1999). Natural killer cells in relapsing-remitting MS: effect of treatment with interferon beta-1B. Neurology 52, 351–359 10.1212/WNL.52.2.351 9932956

[B40] Kent S. J. Karlik S. J. Cannon C. Hines D. K. Yednock T. A. Fritz L. C. (1995). A monoclonal antibody to alpha 4 integrin suppresses and reverses active experimental allergic encephalomyelitis. J. Neuroimmunol. 58, 1–10 10.1016/0165-5728(94)00165-K 7730443

[B41] Kieseier B. C. Stuve O. (2011). A critical appraisal of treatment decisions in multiple sclerosis – old versus new. Nat. Rev. Neurol. 7, 255–262 10.1038/nrneurol.2011.41 21467994

[B42] Korn T. Magnus T. Toyka K. Jung S. (2004). Modulation of effector cell functions in experimental autoimmune encephalomyelitis by leflunomide – mechanisms independent of pyrimidine depletion. J. Leukoc. Biol. 76, 950–960 10.1189/jlb.0504308 15328336

[B43] Korn T. Toyka K. Hartung H. P. Jung S. (2001). Suppression of experimental autoimmune neuritis by leflunomide. Brain 124, 1791–1802 10.1093/brain/124.9.1791 11522581

[B44] Kowarik M. C. Pellkofer H. L. Cepok S. Korn T. Kumpfel T. Buck D. (2011). Differential effects of fingolimod (FTY720) on immune cells in the CSF and blood of patients with MS. Neurology 76, 1214–1221 10.1212/WNL.0b013e3182143564 21464424

[B45] Lim S. Y. Constantinescu C. S. (2010). Current and future disease-modifying therapies in multiple sclerosis. Int. J. Clin. Pract. 64, 637–650 10.1111/j.1742-1241.2009.02261.x 20456216

[B46] MacEwan D. J. (2002). TNF ligands and receptors – a matter of life and death. Br. J. Pharmacol. 135, 855–875 10.1038/sj.bjp.0704549 11861313 PMC1573213

[B47] Manda G. Neagu M. Constantin C. Neagoe I. Codreanu C. (2009). Preliminary study on the immunologic background of good clinical outcome in rheumatoid arthritis patients after one month therapy with leflunomide. Rheumatol. Int. 29, 937–946 10.1007/s00296-008-0802-6 19096851

[B48] Martin J. F. Perry J. S. Jakhete N. R. Wang X. Bielekova B. (2010). An IL-2 paradox: blocking CD25 on T cells induces IL-2-driven activation of CD56(bright) NK cells. J. Immunol. 185, 1311–1320 10.4049/jimmunol.1000802 20543101 PMC3085179

[B49] Martin R. (2012). Anti-CD25 (daclizumab) monoclonal antibody therapy in relapsing-remitting multiple sclerosis. Clin. Immunol. 142, 9–14 10.1016/j.clim.2011.10.008 22284868

[B50] Martinez-Rodriguez J. E. Lopez-Botet M. Munteis E. Rio J. Roquer J. Montalban X. (2011). Natural killer cell phenotype and clinical response to interferon-beta therapy in multiple sclerosis. Clin. Immunol. 141, 348–356 10.1016/j.clim.2011.09.006 21992960

[B51] Martinez-Rodriguez J. E. Saez-Borderias A. Munteis E. Romo N. Roquer J. Lopez-Botet M. (2010). Natural killer receptors distribution in multiple sclerosis: relation to clinical course and interferon-beta therapy. Clin. Immunol. 137, 41–50 10.1016/j.clim.2010.06.002 20580616

[B52] Merrill J. Jondal M. Seeley J. Ullberg M. Siden A. (1982). Decreased NK killing in patients with multiple sclerosis: an analysis on the level of the single effector cell in peripheral blood and cerebrospinal fluid in relation to the activity in the disease. Clin. Exp. Immunol. 47, 419–430 6176377 PMC1536515

[B53] Miller D. H. Khan O. A. Sheremata W. A. Blumhardt L. D. Rice G. P. Libonati M. A. (2003). A controlled trial of natalizumab for relapsing multiple sclerosis. N. Engl. J. Med. 348, 15–23 10.1056/NEJMoa020696 12510038

[B54] Morandi B. Bramanti P. Bonaccorsi I. Montalto E. Oliveri D. Pezzino G. (2008). Role of natural killer cells in the pathogenesis and progression of multiple sclerosis. Pharmacol. Res. 57, 1–5 10.1016/j.phrs.2007.11.003 18182304

[B55] Moretta A. (2002). Natural killer cells and dendritic cells: rendezvous in abused tissues. Nat. Rev. Immunol. 2, 957–964 10.1038/nri956 12461568

[B56] Nayak R. C. Jacolik R. F. (2012). “CD16+/perforin+ lymphocytes (NK cells) regulate autoimmunity in multiple sclerosis by cytolysis of antigen laden cells in the peripheral blood (abstract #178),” Presented at The Society for Natural Immunity Meeting, Asilomar. Available at: http://nk2012.wordpress.com/abstracts/

[B57] Neighbour P. A. Grayzel A. I. Miller A. E. (1982). Endogenous and interferon-augmented natural killer cell activity of human peripheral blood mononuclear cells in vitro. Studies of patients with multiple sclerosis, systemic lupus erythematosus or rheumatoid arthritis. Clin. Exp. Immunol. 49, 11–21 6181920 PMC1536668

[B58] Nielsen N. Odum N. Urso B. Lanier L. L. Spee P. (2012). Cytotoxicity of CD56(bright) NK cells towards autologous activated CD4+ T cells is mediated through NKG2D, LFA-1 and TRAIL and dampened via CD94/NKG2A. PLoS ONE 7:e31959 10.1371/journal.pone.0031959 22384114 PMC3284517

[B59] Nordqvist C. (2012). Aubagio (teriflunomide) approved for multiple sclerosis treatment, FDA. Medical News Today. Available at: http://www.medicalnewstoday.com/articles/250293.php

[B60] Oger J. Kastrukoff L. F. Li D. K. Paty D. W. (1988). Multiple sclerosis: in relapsing patients, immune functions vary with disease activity as assessed by MRI. Neurology 38, 1739–1744 10.1212/WNL.38.11.1739 3185908

[B61] Perini P. Wadhwa M. Buttarello M. Meager A. Facchinetti A. Thorpe R. (2000). Effect of IFNbeta and anti-IFNbeta antibodies on NK cells in multiple sclerosis patients. J. Neuroimmunol. 105, 91–95 10.1016/S0165-5728(00)00196-X 10713368

[B62] Perry J. S. Han S. Xu Q. Herman M. L. Kennedy L. B. Csako G. (2012). Inhibition of LTi cell development by CD25 blockade is associated with decreased intrathecal inflammation in multiple sclerosis. Sci. Transl. Med. 4, 145ra106 10.1126/scitranslmed.3004140 22855463 PMC3846177

[B63] Perussia B. Trinchieri G. Jackson A. Warner N. L. Faust J. Rumpold H. (1984). The Fc receptor for IgG on human natural killer cells: phenotypic, functional, and comparative studies with monoclonal antibodies. J. Immunol. 133, 180–189 6233371

[B64] Planas R. Jelcic I. Schippling S. Martin R. Sospedra M. (2012). Natalizumab treatment perturbs memory- and marginal zone-like B-cell homing in secondary lymphoid organs in multiple sclerosis. Eur. J. Immunol. 42, 790–798 10.1002/eji.201142108 22144343

[B65] Putzki N. Baranwal M. K. Tettenborn B. Limmroth V. Kreuzfelder E. (2010). Effects of natalizumab on circulating B cells, T regulatory cells and natural killer cells. Eur. Neurol. 63, 311–317 10.1159/000276400 20453514

[B66] Ransohoff R. M. (2007). Natalizumab for multiple sclerosis. N. Engl. J. Med. 356, 2622–2629 10.1056/NEJMct071462 17582072

[B67] Romagnani C. Juelke K. Falco M. Morandi B. D’Agostino A. Costa R. (2007). CD56brightCD16- killer Ig-like receptor-NK cells display longer telomeres and acquire features of CD56dim NK cells upon activation. J. Immunol. 178, 4947–4955 17404276 10.4049/jimmunol.178.8.4947

[B68] Sand K. L. Knudsen E. Rolin J. Al-Falahi Y. Maghazachi A. A. (2009). Modulation of natural killer cell cytotoxicity and cytokine release by the drug glatiramer acetate. Cell. Mol. Life Sci. 66, 1446–1456 10.1007/s00018-009-8726-1 19277466 PMC11131507

[B69] Saraste M. Irjala H. Airas L. (2007). Expansion of CD56Bright natural killer cells in the peripheral blood of multiple sclerosis patients treated with interferon-beta. Neurol. Sci. 28, 121–126 10.1007/s10072-007-0803-3 17603762

[B70] Shi F. D. Takeda K. Akira S. Sarvetnick N. Ljunggren H. G. (2000). IL-18 directs autoreactive T cells and promotes autodestruction in the central nervous system via induction of IFN-gamma by NK cells. J. Immunol. 165, 3099–3104 10975822 10.4049/jimmunol.165.6.3099

[B71] Skarica M. Eckstein C. Whartenby K. A. Calabresi P. A. (2011). Novel mechanisms of immune modulation of natalizumab in multiple sclerosis patients. J. Neuroimmunol. 235, 70–76 10.1016/j.jneuroim.2011.02.010 21550672

[B72] Spits H. Artis D. Colonna M. Diefenbach A. Di Santo J. P. Eberl G. (2013). Innate lymphoid cells – a proposal for uniform nomenclature. Nat. Rev. Immunol. 13, 145–149 10.1038/nri3365 23348417

[B73] Spits H. Di Santo J. P. (2011). The expanding family of innate lymphoid cells: regulators and effectors of immunity and tissue remodeling. Nat. Immunol. 12, 21–27 10.1038/nrm3025 21113163

[B74] Strowig T. Brilot F. Munz C. (2008). Noncytotoxic functions of NK cells: direct pathogen restriction and assistance to adaptive immunity. J. Immunol. 180, 7785–7791 18523242 10.4049/jimmunol.180.12.7785 PMC2575662

[B75] Vaessen L. M. Van Besouw N. M. Mol W. M. Ijzermans J. N. Weimar W. (2006). FTY720 treatment of kidney transplant patients: a differential effect on B cells, naive T cells, memory T cells and NK cells. Transpl. Immunol. 15, 281–288 10.1016/j.trim.2006.02.002 16635750

[B76] Vandenbark A. A. Huan J. Agotsch M. La Tocha D. Goelz S. Offner H. (2009). Interferon-beta-1a treatment increases CD56bright natural killer cells and CD4+CD25+ Foxp3 expression in subjects with multiple sclerosis. J. Neuroimmunol. 215, 125–128 10.1016/j.jneuroim.2009.08.007 19758707

[B77] Veeramani S. Wang S. Y. Dahle C. Blackwell S. Jacobus L. Knutson T. (2011). Rituximab infusion induces NK activation in lymphoma patients with the high-affinity CD16 polymorphism. Blood 118, 3347–3349 10.1182/blood-2011-05-351411 21768303 PMC3179401

[B78] Weber M. S. Hohlfeld R. Zamvil S. S. (2007). Mechanism of action of glatiramer acetate in treatment of multiple sclerosis. Neurotherapeutics 4, 647–653 10.1016/j.nurt.2007.08.002 17920545 PMC7479674

[B79] Wynn D. Kaufman M. Montalban X. Vollmer T. Simon J. Elkins J. (2010). Daclizumab in active relapsing multiple sclerosis (CHOICE study): a phase 2, randomised, double-blind, placebo-controlled, add-on trial with interferon beta. Lancet Neurol. 9, 381–390 10.1016/S1474-4422(10)70033-8 20163990

[B80] Xu W. Fazekas G. Hara H. Tabira T. (2005). Mechanism of natural killer (NK) cell regulatory role in experimental autoimmune encephalomyelitis. J. Neuroimmunol. 163, 24–30 10.1016/j.jneuroim.2005.02.011 15885305

[B81] Yednock T. A. Cannon C. Vandevert C. Goldbach E. G. Shaw G. Ellis D. K. (1995). Alpha 4 beta 1 integrin-dependent cell adhesion is regulated by a low affinity receptor pool that is conformationally responsive to ligand. J. Biol. Chem. 270, 28740–28750 10.1074/jbc.270.48.28740 7499396

[B82] Yong V. W. (2002). Differential mechanisms of action of interferon-beta and glatiramer aetate in MS. Neurology 59, 802–808 10.1212/WNL.59.6.802 12349849

[B83] Zhang B. Yamamura T. Kondo T. Fujiwara M. Tabira T. (1997). Regulation of experimental autoimmune encephalomyelitis by natural killer (NK) cells. J. Exp. Med. 186, 1677–1687 10.1084/jem.186.10.1677 9362528 PMC2199138

